# Microfluidic co-culture system for synaptically segregated neural networks to explore astrocyte-driven neural pathology

**DOI:** 10.1038/s41378-026-01187-3

**Published:** 2026-05-14

**Authors:** Yiing C. Yap, Ruth E. Musgrove, Michael C. Breadmore, Rosanne M. Guijt, Richard Wilson, Graeme Wertheimer, Anna E. King, Tracey C. Dickson

**Affiliations:** 1 Menzies Institute for Medical Research, University of Tasmania, Hobart, TAS 7000 Australia; 2 ACROSS, School of Natural Science, University of Tasmania, Hobart, TAS 7000 Australia; 3 Centre for Regional and Rural Futures, Deakin University, Geelong, VIC 3220 Australia; 4 Central Science Laboratory, University of Tasmania, Hobart, TAS 7000 Australia; 5 School of Medicine and Public Health, University of Newcastle, Callaghan, NSW 2308 Australia; 6 Wicking Dementia Research and Education Centre, University of Tasmania, Hobart, TAS 7000 Australia

**Keywords:** Nanoscience and technology, Microfluidics

## Abstract

Investigating astrocyte–neuron communication in the absence of neuron-to-neuron signalling is challenging using traditional culture systems due to the complexity of synaptic networks. To address this, we designed a three-compartment microfluidic co-culture device that fluidically isolates two neuronal populations while permitting astrocyte growth throughout. This design enables assessment of astrocyte-specific contributions to neuropathology between synaptically segregated neurons. The device incorporates ten microchannel banks forming maze-like structures that restrict neurite extension and fluid exchange, while allowing an astrocyte monolayer to infiltrate all compartments. Using this platform, we exposed one neuron–astrocyte population to the excitotoxin kainic acid (KA) and observed neurite degeneration in the adjacent, fluidically isolated neurons connected only via astrocytes. Pre-treatment of astrocytes with the membrane-permeable chelator BAPTA-AM markedly attenuated this effect, implicating calcium in astrocyte-mediated excitotoxicity. This microfluidic system provides a controllable in vitro model of neuron–astrocyte networks, enabling directional connectivity and mechanistic studies of circuit behaviour. Our findings highlight the utility of this platform for exploring intercellular signalling pathways relevant to neurodegenerative disease.

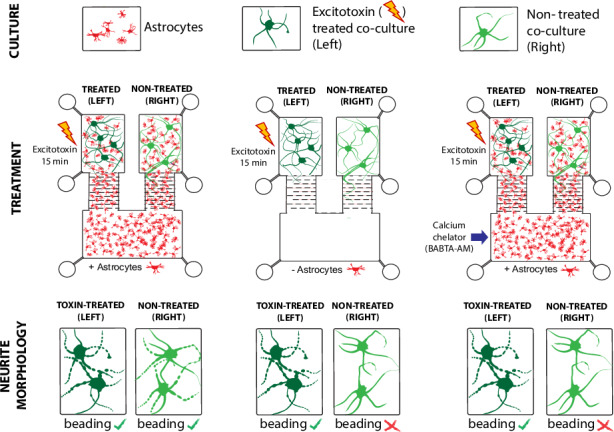

## Introduction

The mammalian central nervous system (CNS) comprises diverse neuronal subtypes and glial cells, including astrocytes, oligodendrocytes, and microglia. Interactions between these cell types are critical for maintaining brain homeostasis and are increasingly implicated in the pathogenesis of neuroinflammatory and neurodegenerative diseases^1–3^. However, the precise roles of neuron–glia and axon–glia interactions in these processes remain poorly understood.

Studying neuron–glia interactions is challenging in vivo due to the brain’s complexity and limited spatial and temporal control over the cellular microenvironment. Conventional in vitro models have failed to isolate the contributions of individual cell types. In contrast, microfluidic platforms offer precise control over cellular environments and connectivity, enabling targeted investigation of specific neuronal features (e.g., soma, axons, dendrites) and neuron–glia interactions^4–7^. While some existing models have explored axon–glia communication and inflammatory signalling, many lack the ability to support direct cell–cell contact and independently manipulate distinct cell populations, both of which are critical for dissecting cellular contributions to CNS pathology.

One of the most widely used microfluidic approaches for neuron–glia co-culture is compartmentalisation, which isolates axons from their cell bodies using microchannels to study axon–glia interactions^4,5^. However, these devices are limited by their inability to control the directionality of neuronal connectivity and to independently manipulate the chemical environments of distinct cell populations. Advanced designs integrating pneumatic valve barriers allow improved control over intercellular communication and treatment, but still do not fully address the need for precise control of neuron–glia connectivity and interaction dynamics^6,7^.

To address these gaps, we developed a microfluidic platform with a maze-structure to co-culture neurons and astrocytes in close contact while maintaining fluidic isolation for cellular analysis. The device features two neuron-containing chambers connected by a central astrocyte-only compartment. This astrocyte compartment is flanked by maze-like structures that restrict axonal growth but permit astrocyte infiltration. We validated our device through the co-culture of primary cortical neurons and astrocytes, demonstrating that the maze-like structures block axonal growth, while being permissive to astrocytes. To our knowledge, this is the first platform to construct a neuronal network with directional connectivity between different populations of neurons and astrocytes. This device enables us to uncouple and independently investigate the role of astrocytes in the temporal and spatial events of neuronal network activity.

To demonstrate the functionality of our co-culture platform, we tested the hypothesis that astrocytes can propagate excitotoxic signals between isolated neuronal populations. Astrocytes actively participate in network communication through intracellular calcium oscillations and gliotransmission^8,9^. Under normal circumstances, astrocytes help to maintain the concentration of excitatory neurotransmitters at the synapse^10,11^. However, in pathological states such as epilepsy, stroke, or traumatic brain injury, dysregulation of this function can lead to excitotoxicity, characterised by excessive neuronal activation^12,13^. Our findings suggest that astrocytes can mediate the spread of excitotoxic pathology via calcium signalling between physically and fluidically segregated neurons. This co-culture platform offers a new tool for dissecting astrocyte-driven mechanisms in CNS dysfunction and provides a model to trial glia-targeted therapeutic strategies that are nearly. This microfluidic system can be readily customised for different cell populations, providing a powerful tool to investigate disease mechanisms and to screen candidate interventions aimed at modulating neuron–glia signalling. Beyond mechanistic studies, the platform’s flexibility supports translational applications, including preclinical testing of neuroprotective compounds and therapies targeting astrocyte-mediated pathology.

## Materials and methods

### Animals

All animal usage was approved by the Animal Ethics Committee of the University of Tasmania (application21736) and conducted according to the Australian Code of Practice for the Care and Use of Animals for Scientific Purposes (2013). Sprague Dawley rats were bred by the University of Tasmania Animal Research Facility and housed in individually ventilated cages with a 12-h light/dark cycle and free access to food and water. Neonatal and embryonic rats of both sexes were used for the preparation of astrocyte and neuron cultures, respectively.

### Fabrication of a co-culture microfluidic device

**Fig. 1:**
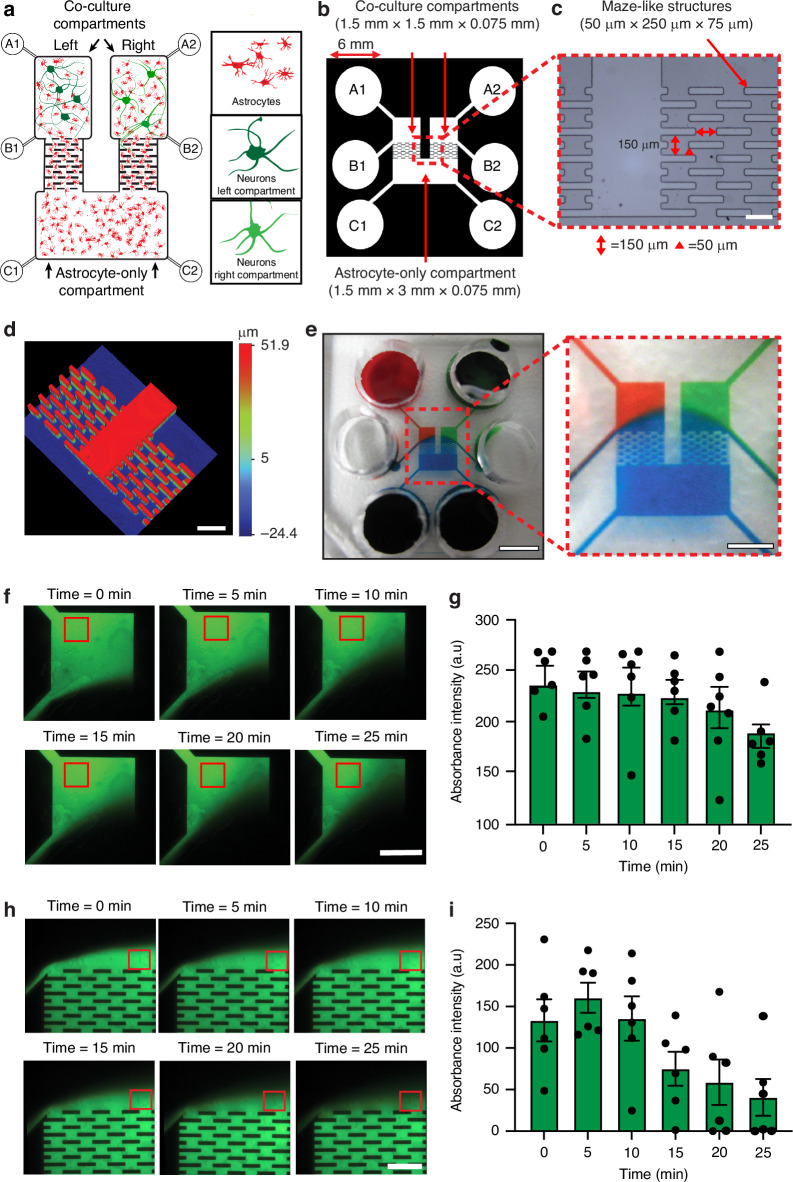
Design and fluidic properties of the microfluidic cell culture device. **a** Schematic of neurons and astrocytes populating the microfluidic device. Left and right co-culture compartments connect to the astrocyte-only compartment by a maze-like structure. The volume in reservoirs A-C hydrostatically controls fluidic isolation of each culture compartment. **b** Scaled schematic of the microfluidic cell culture device, including detailed dimensions. **c** Microscopy image of the maze-like structure with dimensions. Scale bar = 250 µm. **d** A 3D optical profilometry of the maze-like structure, comprising ten segregated banks with dimensions 50 µm × 250 µm × 75 µm. Scale bar = 250 µm. **e** Demonstration of fluidic isolation with coloured dyes in reservoirs A1 (red), A2 (green) and C1 and C2 (blue). Inset magnifies the cell culture compartments, showing fluidic isolation of the left (red dye) and right (green dye) co-culture compartments caused by increased hydrostatic pressure in the astrocyte-only compartment (blue dye). Scale bar = 3 mm; inset 1.5 mm. **f** The left co-culture compartment was filled with fluorescent sodium salt (FI) under fluidic isolation. Fluorescence was measured in the red region of interest (ROI) over 25 min. Scale bar = 750 µm. **g** Absorbance intensity of FI fluorescence (a.u.) in a ROI of the left co-culture compartment quantified over 25 min. **h** The astrocyte-only compartment filled with fluorescent sodium salt (FI) under fluidic isolation. Fluorescence was quantified in the red ROI over 25 min. Scale bar = 500 µm. **i** Quantified changes in FI fluorescence absorbance intensity (a.u.) in a ROI of the top co-culture compartment over 25 min

As illustrated in Fig. 1a–c, the proposed three-compartment microfluidic neuron–astrocyte co-culture platform consists of two neuron/astrocyte compartments (left and right, 1.5 mm × 1.5 mm per compartment) connecting to a third astrocyte-only compartment (bottom, 1.5 mm × 3.5 mm) via maze-like structures. These maze-like structures are designed to inhibit axon growth and prevent synaptic connections between the two neuron populations, whilst allowing astrocyte infiltration across all compartments. A key feature of the device is the capacity to maintain sustained periods of fluidic isolation between the compartments, which is achieved through 6 fluid reservoirs (6 mm diameter) which regulate hydrostatic pressure and fluid flow.

To fabricate the device, a master mould was created on a poly (methyl methacrylate) (PMMA; RS components Pty Limited) substrate (75 mm × 50 mm × 2 mm) using two stages of photolithography. First, the PMMA was thinly coated with a photoresist layer (SU-8 2005, Microchem), soft-baked, overexposed, and then post-baked to form the adhesive surface for the second layer. The process was immediately repeated with a thicker photoresist (SU-8 3025, Microchem) layer, exposed with a high-resolution transparency mask, and developed to generate a second layer that consisted of a maze array of 10 offset banks (dimensions: height = 75 µm, width = 50 µm, length = 250 µm, regular intervals = 50 µm on the Y-axis and spacing between banks = 150 µm on the X-axis), an astrocyte-only compartment (height = 75 µm, width = 1.5 mm, length = 3.5 mm) and two co-culture compartments (height = 75 µm, width = 1.5 mm, length = 1.5 mm). The mould was post-baked, developed and hard-baked. Device replication was facilitated by soft lithography using a pre-polymer curing agent, poly-dimethylsiloxane (PDMS) (10:1 mixture, Sylgard 184, Dow Corning, Inc), followed by curing at 70 °C overnight. Once replicated, the reservoirs for culture media were formed using a biopsy punch (6 mm diameter, Kai Medical).

### Surface profilometry

The PDMS microfluidic devices replicated from the master mould were examined under an optical profiler microscope Wyko NT9100 (Veeco Instrument Inc.) to verify channel dimensions and assess the fidelity of the maze-like structures formed during the replication process. Optical profiler imaging was analysed by using dual LED light optical profiling in the vertical scanning interferometry (VSI) using a 5× objective.

### Pre-culture assembly and preparation of the microfluidic device

Microfluidic devices were cleaned using adhesive tape and washed in 70% ethanol overnight before being irradiated with a UV light for 30 min. The devices were irreversibly bonded onto sterile glass coverslips (24 × 24 mm^2^, Thermo Fisher Scientific) using a handheld air plasma unit (Electro Technic Products Inc). Bonded devices were then coated with 0.001% poly-L-lysine (PLL) in 0.01 M Phosphate Borate Buffer (PBS) to promote cell attachment, and incubated (37 °C, 5% CO_2_) for 24 h prior to washing with sterile milli-Q^®^ and addition of appropriate cell culture media.

Prior to the plating of primary cortical neurons, devices were filled with neuron growth media (NGM) containing Neurobasal™ supplemented with 2% v/v B27, 0.5 mM glutamax, 25 µM L-glutamic acid and 1% v/v penicillin/streptomycin (all from Thermo Fisher Scientific). Prior to plating astrocytes, the devices were filled with glial growth media (GGM) containing DMEM (Thermo Fisher Scientific) supplemented with 10% v/v FBS and 1% penicillin/streptomycin. For the co-culture of neurons with astrocytes, astrocytes were grown in the devices for one week with GGM, 24 h prior to the addition of neurons, media were replaced with modified NGM supplemented with 10% v/v FCS (Thermo Fisher Scientific).

### Confirmation of fluidic isolation

The efficiency of fluidic isolation was indirectly measured by observing changes in the fluorescence intensity of fluorescein sodium salt (FI) loaded into the left co-culture compartment. FI was diluted to 1 mg/mL in 0.01 M PBS and added to reservoir A1. Fluidic isolation was achieved by maintaining the fluid volume of both the top wells (reservoirs A1 and A2) of the co-culture compartments at a lower level (10 µL total volume) than that of the astrocyte-only compartment (reservoirs C1 and C2; 20 µL), while the middle compartment (reservoirs B1 and B2) remained empty. The fluorescent signal was imaged over 25 min using a Nikon Eclipse TI-U inverted fluorescence microscope, equipped with NIS-Elements BR 3.10 software (Melville) and fitted with a high-definition colour charge-coupled device (CCD) camera (Nikon). Imaging was performed under a 10× objective with a filter cube (Semrock, Rochester) that consisted of an excitation band-pass filter (488 ± 10 nm), emission filter (520 ± 10 nm), and dichroic mirror. The region of interest (ROI) was defined, and changes in fluorescence intensity (a.u.) were measured using FIJI (NIH). To test the fluidic isolation of the astrocyte-only compartment, we reversed the hydrostatic pressure difference by adding 10 µL to the co-culture compartments (reservoirs A1 and A2) and introduced localised FI solution to reservoirs C1 and C2. We then imaged the left-hand side of the co-culture compartment and the maze-like structure within the device. In addition, we also analysed the fluorescent absorbance of the solution from each compartment using Fluostar Optima automated plate reading fluorometer (BMG LABTECH), set to 460 nm excitation and 515 nm emission detection for FI following 15 min localised FI treatment to the reservoir A1 compartment.

### Co-culture of astrocytes and cortical neurons

Our microfluidic devices were populated with primary astrocytes, cortical neurons, or neuron/astrocyte co-cultures, according to the timeline outlined in Fig. S1. To obtain astrocyte cultures, primary mixed glial cells were prepared from the cortices of postnatal day 2–3 (P2–3) rat pups, as described previously^14^, with minor modifications. The meninges were removed, and cortical tissue was dissociated in 0.0125% trypsin at 37 °C for 5 min. Trypsin was inactivated with GGM, and the tissue was mechanically dissociated. The cell suspension was filtered through a 70 µm gauze and centrifuged (5 min, 300 rcf, room temperature). Cell pellets were dissociated in fresh GGM and plated into T75 flasks pre-coated with 0.001% PLL in PBS. At 7–8 days, when confluency was reached, the flask was shaken at 200 rpm, 37 °C, overnight to separate non-astrocytic glia from the astrocyte layer. Detached glial cells were removed, and astrocytes were harvested using 0.05% trypsin/EDTA (Sigma). Cells were loaded into the astrocyte-only and co-culture compartments of the microfluidic devices through reservoirs B1, B2, C1 and C2 at a density of 5 × 10^6^ cells. GGM was replenished every 3 days.

Cortical neurons were prepared from embryonic day 18 (E18) Sprague Dawley rats as described previously^15^. After dissection, cortices were dissociated in 0.0125% trypsin at 37° for 5 min, washed in modified NGM and triturated. Reservoirs C1 and C2 were filled with 20 µL of pre-warmed modified NGM. Cortical neurons (6 × 10^6^) were suspended in 10 µL of modified NGM and were then seeded into the co-culture compartments through reservoirs A1 and A2. By maintaining the media level in the astrocyte-only compartment higher than the other compartments, hydrostatic pressure prevented the dissociated neurons from fluidically entering the maze. After seeding, the neurons were incubated at 37 °C, 5% CO_2_, for 5 min to allow adherence_._ All compartments were then slowly filled with pre-warmed modified NGM. Cultures were grown at 37 °C, 5% CO_2_ and modified NGM was replaced 3 times per week.

To prepare kainic acid (KA)-conditioned media, primary cortical neurons were cultured as described above, with dissociated cells plated at a density of 3 × 10⁴ cells per 12 mm round coverslip. Cultures were maintained at 37 °C with 5% CO₂, and NGM was replaced 3 times per week. At 7 days in vitro (DIV), 1 mM KA was applied to the neuronal monolayers for 15 min at 37 °C, 5% CO₂. Monolayers were then washed with fresh NGM and incubated for an additional 6 h under the same conditions. The resulting KA-conditioned media was collected and applied to neuron/astrocyte co-cultures in the microfluidic devices for 15 min.

### Pharmacological manipulation

KA (1 mM, Sigma-Aldrich) or the membrane-permeable calcium chelator BAPTA-AM (BA; 1 µM; 1,2-bis-(2-aminophenoxy)ethane-N,N,N′,N′-tetraacetic acid tetra(acetoxymethyl) ester, Santa Cruz Biotechnology) or Dimethyl sulfoxide (DMSO; Sigma) vehicle control was prepared in modified NGM and pre-warmed to 37 °C prior to application. Alone KA treatment was applied to the left co-culture compartment at 7 DIV. Fluidic isolation of the treated left co-culture compartment was achieved as described above. BA was applied to the astrocyte-only compartment for 15 min at 7 DIV. Treated cultures were maintained under fluidic isolation in standard growth conditions for 5 or 15 min, followed by 3 washes with fresh modified NGM.

### Protein sample preparation for mass spectrophotometry

Proteins were harvested from the left co-culture, right co-culture, and astrocyte-only compartments of the devices at 7 DIV of neuron/astrocytes co-culture. Cells were washed 3 times with cold PBS, then lysed in lysis buffer containing urea (7 M, Sigma), thiourea (2 M, Sigma), Tris-HCl (30 mM, Sigma), dithiothreitol (DTT, 10 mM, Sigma) and EDTA-free protease inhibitors (Roche). Cell lysates were sonicated on ice for 3 cycles of 15 s pulse with 5 s intervals. Cell lysates were gently mixed on a rotary suspension mixer at 4 °C for 16 h and centrifuged at 16,000 *g* for 30 min. The supernatant was collected in protein LoBind tubes (Eppendorf). Protein concentration was quantified using the Pierce 660 nm Protein Assay (Thermo Fisher Scientific) according to the manufacturer’s instructions. For each sample P, 20 µg of protein was sequentially reduced overnight at 4 °C using DTT (10 mM, Sigma) and alkylation with iodoacetamide (IAA, 50 mM, Sigma) for 2 h at room temperature in the dark. The samples underwent single-pot, solid-phase-enhanced sample preparation (SP3), followed by digestion with 1.2 µg proteomic-grade trypsin/rLysC (Promega) overnight at 37 °C.

### Mass spectrometry (MS)

Protein samples were analysed by nanoflow high-performance liquid chromatography–MS/MS using an Ultimate 3000 RSLC nano system (Thermo Fisher Scientific) coupled with a Q-Exactive HF mass spectrometer fitted with a nanospray Flex ion source (Thermo Fisher Scientific) and controlled using Xcalibur software (version 4.3). Approximately 1 µg of sample was injected and separated using a 120-min segmented gradient by preconcentration onto a 20 mm × 75 µm PepMap 100 trapping column (3 µm C18), then separation on a 250 mm × 75 µm PepMap 100 RSLC column (2 µm C18) at a flow rate of 300 nL/min and held at 35 °C. MS Tune software (version 2.9) parameters used for data acquisition were as follows: 2.0 kV spray voltage, S-lens RF level of 60 and heated capillary set to 250 °C. MS1 spectra (390–1240 m/z) were acquired at a scan resolution of 120,000 in profile mode with an automatic gain control (AGC) target of 3 × 10^6^ and followed by sequential MS2 scans across 26 data independent acquisition (DIA) × 25 single atomic mass unit (amu) windows over the range of 397.5–1027.5 m/z, with 1 amu overlap between sequential windows. MS2 spectra were acquired in centroid mode at a resolution of 30,000 using an AGC target of 1 × 10^6^, maximum injection time of 55 ms and normalised collision energy of 27 eV.

### MS raw data processing and statistical analysis

The DIA MS raw files were processed using Spectronaut software (version 18, Biognosys AB). A project-specific library comprising 6308 protein groups was generated using the Pulsar search engine to search the DIA MS1 spectra against the *Rattus norvegicus (Rat)*, UniProt reference proteome UP000002494, concatenated with common contaminants (comprising 22,898 entries, downloaded in November 2023). With the exception that single-hit proteins were excluded, default (Biognosys factory) settings were used for both spectral library generation and DIA data extraction. For library generation, these included N-terminal acetylation and methionine oxidation as variable modifications and cysteine carbamidomethylation as a fixed modification, up to two missed cleavages allowed, and peptide, protein and peptide spectrum match thresholds set to 0.01. Mass tolerances were based on first-pass calibration and extensive calibration, for the calibration and main searches, respectively, with correction factors set to 1 at the MS1 and MS2 levels. Targeted searching of the library based on ion current extraction deployed dynamic retention time alignment with a correction factor of 1.

Quantification was based on the MaxLFQ algorithm for the derivation of inter-run peptide ratios, followed by cross-run normalisation based on median peptide intensity. Differential abundance of proteins of astrocytes/neuron culture and astrocytes-only culture in different compartments was determined using one-way ANOVA followed by post hoc test with unpaired two-tailed Student’s *t*-test with a permutation-based false discovery rate set at 0.05 and S0 = 0.1 to exclude proteins with very small difference between means (Perseus software version 2.1.2.0, http://www.coxdocs.org/doku.php?id=perseus:start). Protein interaction networks were identified using STRING (version 12.0, http://www.string-db.org/). Identified proteins that were significantly increased or decreased in abundance in neuron/astrocytes co-cultures compared to astrocyte-only cultures in different compartments were uploaded to DAVID Bioinformatics Resource 6.8 (https://david.ncifcrf.gov/), to retrieve the Reactome pathway and the Gene ontology (GO) enrichment analysis for molecular function, cellular compartment and biological process with *P* < 0.05 after Benjamini–Hochberg correction were considered significant. The mass spectrometry proteomics data have been deposited to the ProteomeXchange Consortium via the PRIDE^16^ partner repository with the dataset identifier PXD055523.

### Immunocytochemistry and confocal analysis

Neuron-only or neuron–astrocyte cultures were fixed with 4% (w/v) paraformaldehyde (Sigma) in PBS for 30 min at room temperature and washed with 0.01 M PBS. Fixed cells were permeabilised with 0.3% v/v Triton X-100 in 0.01 M PBS for 30 min prior to blocking in 0.01 M PBS containing Triton X-100 (0.3% v/v; Sigma) and BSA (0.5% w/v; Sigma) for 30 min. Cultures were immunolabelled with primary antibodies diluted in blocking solution: β-III tubulin (mouse, Promega) for neuron-only cultures, and βIII tubulin and GFAP (rabbit, Dako) for neuron–astrocyte co-cultures. Primary antibody incubation was performed for 1 h at room temperature, followed by overnight incubation at 4 °C. After washing 3 times with 0.01 M PBS for 10 min each, cultures were incubated with Alexa Fluor-conjugated secondary antibodies (1:1000; Thermo Fisher Scientific) in the dark for 2 h at room temperature. Samples were then washed, mounted onto glass slides, and allowed to air dry. Fluorescent images were captured on an upright UltraVIEW Spinning Disk confocal microscope with 20× and 40× objectives, using Velocity software (PerkinElmer).

For quantitative analysis, axon penetration through the maze-like structures was assessed by expressing the number of axons between each bank as a percentage of the total number of axons between banks 1 and 2. The mean fluorescence intensity of GFAP labelling between each bank of the maze was measured from a single plane image. To assess excitotoxic pathology, images were acquired from a minimum of 3 random sites within each co-culture compartment. Three confocal images were analysed from each culture as maximum intensity projections. The percentage of beaded neurites, defined as the appearance of “beads on string” or neurite varicosities, was scored in two 50 µm × 50 µm ROIs created within each image using FIJI (NIH). Values were expressed as a percentage of beaded neurites within each ROI.

### Calcium imaging

To assess the effect of BA and KA on astrocytic calcium levels, astrocytes in the astrocyte-only compartment were loaded with the membrane-permeable calcium reagent Fluo-4 direct. Fluo-4 was added to cell culture media and incubated for at least 1 h at 37 °C, 5% CO_2_ prior to imaging, according to the manufacturer’s instructions. Images were captured every 2 s for 2 min using a motorised inverted microscope Nikon Eclipse Ti2 (Nikon Instrument Inc, 20× objectives). After 1 min of baseline recording, KA (final concentration of 1 mM) was spiked (1:1000) into the reservoir A1 to treat cells in the left co-culture compartment. BA was applied to the astrocyte-only compartment (1:1000) through reservoirs C1 and C2 to achieve a final concentration of 1 µM. For control, Fluo-4 reagent was added to cultures in the absence of drug treatment. Imaging was performed as per the treated cultures, with representative images and measurements shown in Fig. S2a and b. ROIs were drawn around individual astrocyte somas, and changes in fluorescence intensities were analysed with FIJI (NIH). Graphs representing calcium dynamics were calculated using the formula: (F-F_0_)/F_0_, where F is defined as the fluorescence intensity within a specified ROI of one cell at time *t*, and *F*_0_ is the baseline fluorescence intensity at time t.

### Statistical analysis

Statistical tests were performed in Prism 8.0 (GraphPad). Data were derived from a minimum of 3 separate culture repeats. All graphical data is presented as means ± standard error of the mean (SEM). For comparison between groups, unpaired, two-tailed *t*-tests were used, with a significance threshold of *p* < 0.05. For comparisons involving more than 2 groups, one-way ANOVA followed by Tukey’s post hoc test was performed, with *p* < 0.05 considered statistically significant.

## Results

### Fabrication of the microfluidic co-culture device

We designed a new cell culture device to study the role of astrocytes in communication between physically and chemically isolated neuron populations. To fabricate this device, a master mould was created on a PMMA substrate using a two-layer SU-8 photolithography process. The microfluidic co-culture platform was then fabricated in PDMS, a biocompatible, transparent, gas-permeable polymer, following well-established replica moulding procedures^17^. Microscope (Fig. 1c) and 3D optical profiler (Fig. 1d) image of the PDMS device demonstrated that the replication of the platform was successfully performed by casting the biocompatible polymer PDMS from the master mould. The device incorporates a maze array composed of multiple offset banks, each measuring 75 µm in height, 50 µm in width, and 250 µm in length. Banks are spaced at regular intervals of 50 µm along the Y-axis, with adjacent banks separated by 150 µm along the X-axis (Fig. 1c, d).

### Fluidic isolation within different compartments of the device

Fluidic isolation within the device is critical for controlling the environment of each cell population within the network. To demonstrate this, coloured dyes were placed in reservoirs labelled A1 and A2 (red and green; 10 µL). A larger volume of fluid was added to reservoirs C1 and C2 (blue; 20 µL), increasing the liquid level in the astrocyte-only side (Fig. 1e). This hydrostatic pressure difference generated a small positive flow from the astrocyte-only compartment toward the empty reservoirs B1 and B2, which exceeded the opposing flow from reservoirs A1 and A2 toward reservoirs B1 and B2. To assess the capacity of our device to maintain fluidic isolation between the left and right co-culture compartments, FI was introduced, and changes in fluorescent intensity were measured over time (Fig. 1e). The left co-culture compartment remained fluidically isolated for 25 min (Fig. 1f, g). Localised addition of FI in reservoir A1 did not produce measurable fluorescence in the right co-culture astrocyte-only compartment or within 15 min, indicating effective fluidic isolation. This was supported by absorbance measurements at 485 nm, which were markedly higher in A1 (1.763 ± 0.028) compared to A2 (0.001 ± 0.004), C1 (0.020 ± 0.011), and C2 (0.024 ± 0.015) (*p* < 0.001; Fig. S3).

Similarly, we reversed the hydrostatic pressure to isolate the astrocyte-only compartment by increasing the volume in the co-culture reservoirs (A1 and A2) by 10 µL compared with the astrocyte-only reservoirs (C1 and C2). We applied 20 µL of the FI solution to reservoirs C1 and C2, and 30 µL of PBS solution to the top reservoirs A1 and A2, while the middle reservoirs B1 and B2 remained empty. Under these conditions, the FI solution was contained within the bottom astrocyte-only compartment. However, some FI solution was observed in the inner corner of the top co-culture compartment (Fig. 1h). The fluorescence intensity of FI within this inner corner region decreased over time as the hydrostatic pressure differential caused a gradual flow from the reservoirs A1, A2 toward C1, C2 of the bottom astrocyte-only compartment (Fig. 1h, i). This reflects the transition of a sharp interface to a more diffuse boundary, indicative of reduced flow rates and increased diffusion. Importantly, no fluorescence was detected in reservoirs A1 and A2 for at least 25 min following FI addition to reservoirs C1, C2 (data not shown).

### Growth of astrocytes throughout the compartments

**Fig. 2:**
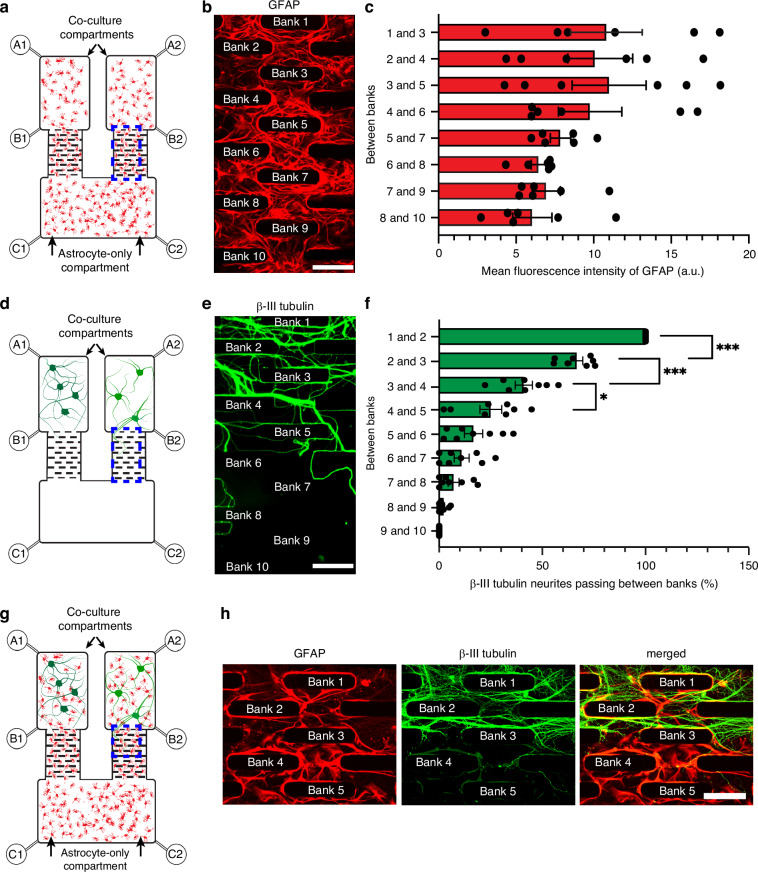
Efficacy of the microfluidic cell culture device to prevent synaptic connections between two fluidically isolated neuron populations whilst permitting astrocyte proliferation. **a** Schematic of microfluidic device populated with astrocytes. The blue box represents the area captured in (**b**). **b** Astrocytes labelled for GFAP (red) within the maze-like structure at 7 DIV. Scale bar = 200 µm. **c** GFAP fluorescence between each bank of the maze-like structure at 7 DIV. *n* = 3 (mean ± SEM). **d** Schematic of a microfluidic device with neurons only in the co-culture compartments. Blue box represents the area captured in (**e**). **e** Neurites from the co-culture compartment, immunolabelled for β-III tubulin (green), were unable to penetrate through the 10 banks of the maze-like structure at 10 DIV. Scale bar = 150 µm. **f** Efficacy of each bank of the maze-like structure to prevent the passage of β-III tubulin immunoreactive neurites at 10 DIV. *p < 0.05; ***p < 0.001; one-way ANOVA with Tukey’s post hoc test. *n* = 3 (mean ± SEM). **g** Schematic diagram of the microfluidic device populated with neurons and astrocytes in the co-culture compartments, and astrocytes in the astrocyte-only compartment. The blue box represents the area captured in (**h**). **h** Immunocytochemical labelling of neurites (β-III tubulin; green) between banks 1 and 4 of the maze-like structure at 7 DIV, when co-cultured with astrocytes (GFAP; red). Scale bar = 125 µm

Prior to co-culture with neurons, we first examined whether astrocytes could form a confluent layer throughout the microfluidic device. An astrocyte suspension (5 × 10^6^ cells/mL) was loaded through reservoirs B1, B2, C1 and C2, to infiltrate all compartments (Fig. 2a). By 7 DIV, astrocytes infiltrated all compartments and formed a confluent monolayer throughout the device. Quantification of GFAP fluorescence intensity between each maze bank confirmed that the maze-like structure did not impede astrocyte infiltration (Fig. 2b, c).

### Restricted neuronal growth

Having achieved confluent growth of astrocytes throughout the device, we next assessed whether the maze-like structure could effectively inhibit synaptic connectivity between the two neuronal populations (Fig. 2d, e). The maze-like structure consisted of 10 offset banks to inhibit neurite extension from the co-culture compartments into the astrocyte-only compartment. To assess its efficacy, we calculated the proportion of β-III tubulin immunopositive neurites crossing each bank relative to the total number entering at bank 1 (10 DIV). Each of the first seven banks corresponded to a 37.45% ± 1.38 decrease in neurite number, with statistically significant decreases (mean = 38.97% ± 2.51, *P* < 0.05) between banks 2 and 3, banks 3 and 4, and banks 4 and 5. By bank 7, less than 10% (7.00% ± 2.65) of the neurites remained; fewer than 2% extended past bank 8, and no neurites crossed bank 9 (Fig. 2f). A maze-like structure of 10 banks was therefore integrated into the device.

To prevent neuronal entry into the astrocyte-only compartment during plating, hydrostatic pressure was adjusted by adding 20 µL of medium into reservoirs labelled C1 and C2 (astrocyte-only compartment), and a lesser volume of 10 µL of cortical neuron suspension (6.5 × 10^6^ cells/mL), reservoirs A1 and A2 (co-culture compartments) (Fig. 2d). Although neurites entered the maze, none progressed beyond bank 9 by 10 DIV (Fig. 2e, f). These data confirmed that the 10-bank maze prevents neurite extension into the astrocyte-only compartment for at least 10 DIV.

### Constructing complex networks between astrocytes and neurons

To establish complex networks within the microfluidic device, astrocytes and neurons were co-cultured in the left and right co-culture compartments. Astrocytes (5 × 10^6^ cells/mL) were first loaded through reservoirs B1, B2, C1, and C2. By 7 DIV, the astrocytes immunolabelled for GFAP (red) reached confluence throughout the device. Primary cortical neurons (6.5 × 10^6^ cells/mL; 10 µL) were then seeded into each co-culture compartment via reservoirs A1 and A2 (Fig. 2g). To prevent the suspended neurons from flowing into the astrocyte-only compartment, low hydrostatic pressure was created by filling reservoirs C1 and C2 with 20 µL of media. Neurons were co-cultured on the established astrocyte layer for 7 days. After this period, neurites, immunolabelled for β-III tubulin (green), remained confined to the neuron/astrocyte compartments, not penetrating past bank 5 of the maze structures (Fig. 2h).

### Protein profile analysis from the neuron/astrocytes co-culture compartment and the astrocytes-only compartment

**Fig. 3:**
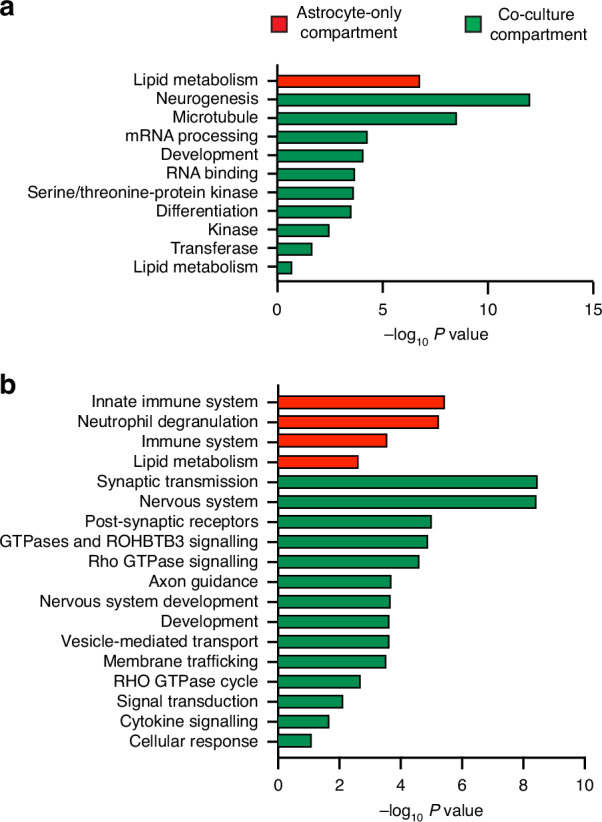
Proteomic profiles of cellular populations in different compartments of the microfluidic device, clustered according to cellular function. **a** Summary of significant differences in proteomic profile between the astrocyte-only (red) and co-culture (green) compartments at 7 DIV. The proteomic profile was functionally categorised by the Gene Ontology (GO) pathway analysis. Significance was determined by an enrichment score of ≥1.3, protein count ≥15 and −log_10_*P* value ≥ 1.3. **b** Summary of significant differences in proteomic profile of the astrocyte-only (red) and co-culture (green) compartments as functionally categorised by the Reactome pathway analysis. Significance was determined by an enrichment score of ≥1.3, protein count ≥15 and −log_10_*P* value ≥ 1.3

To further assess the efficacy of our device in separating cell populations, proteomic analysis was used to compare the co-culture and astrocyte-only compartments. We identified 363 proteins that were significantly (−log_10_*P* value ≥ 5) more abundant in the astrocyte-only compartment when compared to the co-culture compartment. Conversely, 358 proteins were significantly more abundant in neuron/astrocyte co-culture compared to the astrocyte-only culture. Gene Ontology (GO) enrichment analysis of the 358 enhanced proteins in the co-culture compartment was used to group these proteins according to their roles in biological process, molecular functions and as constituents of cellular compartments. The most represented biological processes included mRNA processing, lipid metabolism, differentiation and neurogenesis (Fig. 3a). Enriched molecular functions primarily comprised RNA-binding, kinases, transferases, serine/threonine-protein kinases and developmental processes. RNA-binding proteins play an important role in regulating axon guidance, branching and synaptic structure^18^. The most enriched cellular component was microtubules, the major cytoskeletal component of neurons^19^. In contrast to the neuron/astrocyte co-culture, protein samples from the astrocytes-only compartment showed enrichment of lipid metabolism in the GO terms (Fig. 3a), in line with the metabolic functions of astrocytes, as described by Zhang and colleagues^20^. These data provide further evidence of the efficacy of the microfluidic device in separating cell populations.

The cellular composition of each compartment was further characterised through enrichment analysis of the Reactome pathways^21^. Figure 3b compares the major signalling pathways involved in samples collected from the astrocyte-only and co-culture compartments. Cells in the astrocyte-only compartment were characterised by their involvement in immune response, including the innate immune system (e.g. S100b) and neurotrophil granulation (Fig. 3b, Table S1). Samples from the co-culture compartment were involved in Reactome pathways of axon neuronal system, chemical synapses and neurotransmitter receptors (Fig. 3b). Table S2 show the genes involved in these pathways, including the Synapsin-1 (Syn1), and Calcium/Calmodulin Dependent Protein Kinase II Alpha (Camk2a). We then determined the putative association networks interactions for the genes that encode major pathways through String network analysis for both the co-culture (Fig. S4a) and astrocyte (Fig. S4b).

### Pharmacological treatment in a fluidically isolated environment uncovers the role of astrocytes in the transfer of excitotoxicity

**Fig. 4:**
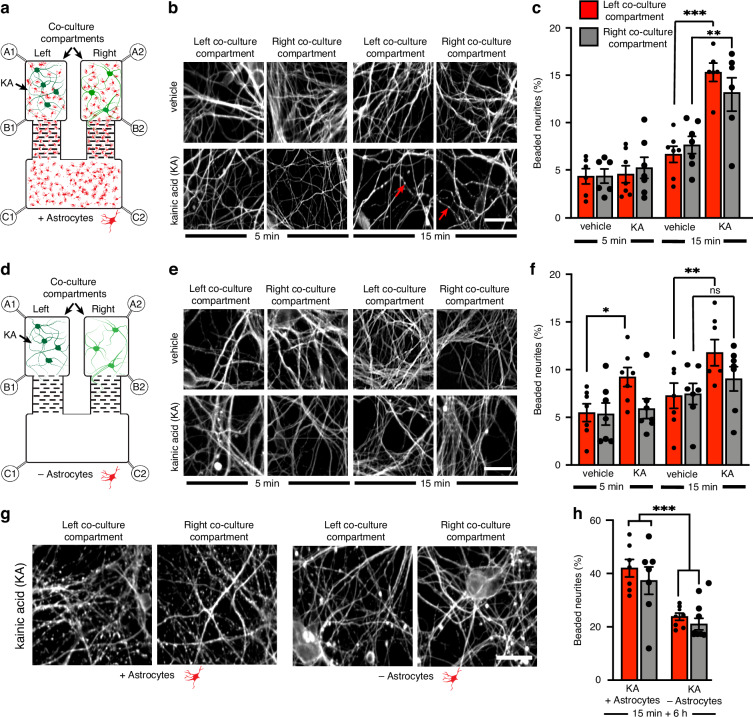
Astrocytes potentiate the loss of neurite integrity in neurons of the right co-culture compartment following kainic acid (KA) treatment to the left co-culture compartment. **a** Microfluidic device schematic, populated with astrocytes and neurons. Arrow signifies KA (1 mM) administration (5 and 15-min) to the left co-culture compartment. **b** Effect of 5 and 15-min of KA treatment on neurite (β-III tubulin) integrity in the left and right co-culture compartments. Arrows indicate beaded axons. Scale bar = 25 µm. c. Beaded axons (%) in ROIs (mm^2^) of each co-culture compartment following KA treatment to the left co-culture compartment in the presence of astrocytes; **p < 0.01; ***p < 0.001; one-way ANOVA with Tukey’s post hoc test. *n* = 3 (mean ± SEM). **d** Microfluidic device populated with neurons without astrocytes. Arrow signifies KA (1 mM) administration (5 and 15-min) to the left co-culture compartment. **e** Effect of 5 and 15-min KA on neurite (β-III tubulin) integrity in each co-culture compartment in the absence of astrocytes. Scale bar = 25 µm. **f** Beaded axons (%) in a ROI (mm^2^) in each co-culture compartment following 5, or 15-min KA treatment in the absence of astrocytes; *p < 0.05; **p < 0.01; one-way ANOVA with Tukey’s post hoc test. *n* = 3 (mean ± SEM). **g** Effect KA treatment on neurite (β-III tubulin) integrity in neurons of each co-culture compartment 6 h after KA treatment (1 mM; 15 min) in the presence vs absence of astrocytes. Scale bar = 25 µm. **h** The effect of astrocytes on the percentage of beaded axons in an ROI (mm^2^) in each co-culture compartment 6 h after 15-min KA treatment; ***p < 0.001; one-way ANOVA with Tukey’s post hoc test. *n* = 3 (mean ± SEM)

Once validated, we used the co-culture platform to investigate the role of astrocytes in the transfer of excitotoxicity between segregated neuronal populations. At 7 DIV of neuron/astrocyte co-culture, Kainic Acid (KA, 1 mM) was applied exclusively to the left co-culture compartment for either 5 or 15 min, under fluidic isolation (Fig. 4a). Neurite integrity (axonal beading and fragmentation) was assessed with β-III tubulin immunoreactivity immediately after treatment (5 or 15 min). Five minutes of KA exposure was not associated with a significant alteration in neurite integrity compared to untreated neurites in the right co-culture compartment. Similarly, no significant differences in neurite integrity were observed between vehicle- and KA- treated cultures after 5 min (Fig. 4b and c). However, after 15 min of KA exposure, there was a significant (*p* < 0.001) increase in the percentage of beaded neurites in the left KA-treated co-culture compartment (15.31% ± 0.97) compared to the vehicle control (6.65% ± 0.86). A comparable and significant (*p* < 0.01) increase was observed in the right untreated co-culture compartment (13.17% ± 1.83) relative to vehicle control (7.63% ± 0.91) (Fig. 4b, c), indicating a transfer of pathology between the fluidically and synaptically isolated neuronal populations via astrocytes.

To further investigate the role of astrocytes in this transfer of pathology, we repeated the experiment using devices cultured with neurons only, in the absence of astrocytes (Fig. 4d). After 7 DIV, the left compartment was treated with KA (1 mM) for 5 or 15 min under fluidic isolation, and neurite morphology was assessed immediately post-treatment. After 5 min of KA exposure, a significant increase (*p* < 0.05) in the percentage of beaded β-III tubulin-positive neurites was observed in the left KA-treated compartment (9.22 ± 1.00%), compared to vehicle-treated controls (5.49 ± 0.93%) (Fig. 4e, f). After 15-min treatment, this increase (*p* < 0.01) was more pronounced (11.78% ± 1.38 vs 7.26% ± 1.32). However, no significant changes in neurite beading were observed in the right untreated compartment compared with vehicle-treated cultures, following either 5- or 15-min KA treatments (Fig. 4e, f). These data indicate that, in the absence of astrocytes, no transfer of pathology occurs between the left and right compartments at these acute time points.

### Investigating the transfer of excitotoxicity pathology following loss of fluidic isolation

Having demonstrated that astrocytes are critical in the transfer of KA-pathology between segregated neuron populations under fluidic isolation, we next aimed to differentiate the role of astrocytes and soluble factors released from neurons in this process. The left co-culture compartment was fluidically isolated during the 15-min KA treatment. Media was then replaced, and cultures were maintained for 6 h without fluidic isolation. At 6 h post-treatment, the percentage of beaded β-III tubulin immunoreactive neurites was significantly higher in both the left (42.00% ± 3.27) and right (37.32% ± 5.16) co-culture compartments compared to vehicle-treated cultures (8.03% ± 1.04 and 7.29% ± 1.28, respectively; *p* < 0.001) (Fig. S5a). These results indicated that a 15-min KA (1 mM) treatment leads to significant neurite degeneration by 6 h post-treatment. Importantly, degeneration was also observed in the right co-culture compartment, which has no synaptic connections with the left compartment and is connected only by the astrocyte population.

To differentiate the roles of astrocytes and soluble factors secreted from neurons in the spread of excitotoxic pathology, we repeated the experiment in devices seeded with neurons in the absence of astrocytes. At 6-h post-treatment, the percentage of beaded neurites was significantly higher in the left (23.81% ± 1.35) and right (21.02% ± 2.17) compartments of KA-treated cultures, relative to their respective vehicle-treated controls (10.16% ± 1.13 and 11.61% ± 1.35; *p* < 0.01) (Fig. S5b). These data indicate that when fluidic isolation is lost, soluble factors can mediate the transfer of excitotoxic pathology between physically separated neuron populations in the absence of astrocytes.

We next aimed to further dissect the contribution of astrocytes and neurons in the spread of excitotoxic pathology. We compared the percentage of beaded β-III tubulin immunoreactive neurites between co-cultures and neuron-only cultures at 6 h following 15-min KA treatment (1 mM) to the left compartment (Fig. 4g, h). In the left KA-treated compartment, the percentage of beaded neurites was significantly higher (*p* < 0.001) in the neuron/astrocyte co-cultures (42.00% ± 3.27) than in neuron-only cultures (23.81% ± 1.35) (Fig. 4g, h), suggesting that astrocytes potentiate KA-induced excitotoxicity. Similarly, neurite beading in the right astrocyte-connected compartment of co-cultures (37.32 ± 5.16%) was significantly greater (*p* < 0.001) than in the right compartment of neuron-only cultures (21.02 ± 2.17%; Fig. 4g, h). These data suggest that astrocytes enhance KA-induced excitotoxicity in neurons and potentiate the toxicity in the right, untreated compartment.

**Fig. 5:**
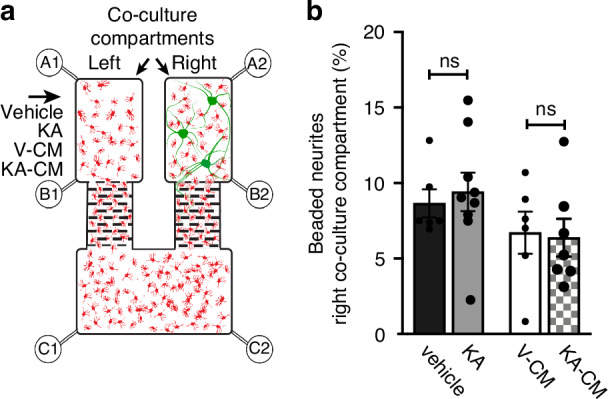
Conditioned media (CM) derived from KA-treated neuronal cultures (KA-CM) did not impair neurite integrity. **a** Schematic diagram of a microfluidic device populated with astrocytes and neurons. The arrow indicates treatments applied to the left co-culture compartment. Conditioned media were derived from vehicle (V-CM) or KA-treated neuronal cultures (KA-CM). **b** Effect of vehicle, KA, V-CM or KA-CM on neurite integrity in the right co-culture compartment; ns non-significant; one-way ANOVA with Tukey’s post hoc test. *n* = 3 (mean ± SEM)

To further characterise the contribution of astrocytes to neuronal pathology, we cultured astrocytes in our microfluidic device, and at 7 DIV co-cultured neurons into the right co-culture compartment, leaving the left compartment with astrocytes only (Fig. 5a). After 7 days of co-culture, astrocytes in the left compartment were treated with 1 mM KA for 15 min under fluidic isolation. Media was then replaced, and neurite degeneration was quantified at 6 h post-treatment in the right co-culture compartment. KA was not associated with an increase in the percentage of beaded β-III tubulin immunoreactive neurites in the right, co-culture compartment (9.41% ± 1.28), compared with vehicle control (8.65% ± 0.93) (Fig. 4b). These data suggest that astrocytes cannot transfer KA-induced excitotoxicity independently from neurons.

We therefore aimed to determine whether a soluble factor released by neurons is required to engage astrocytes in the spread of pathology. We collected conditioned media from neuron-only cultures treated with vehicle (V-CM) or 1 mM KA (KA-CM) for 15 min. Media was replaced and harvested 6 h post-treatment. KA-CM was then applied to astrocytes in the left compartment for 15 min under fluidic isolation, and neurite integrity was assessed in the right co-culture compartment at 6 h post-treatment. No significant difference in the percentage of beaded β-III tubulin immunoreactive neurites was observed between KA-CM-treated (6.38 ± 1.25%) and V-CM (6.72 ± 1.40%) conditions (Fig. 5b). These data suggest that the transfer of excitotoxic pathology through astrocytes is not solely dependent on soluble factors released from neuron cultures in response to KA.

### Live-cell calcium imaging in the microfluidic device

**Fig. 6:**
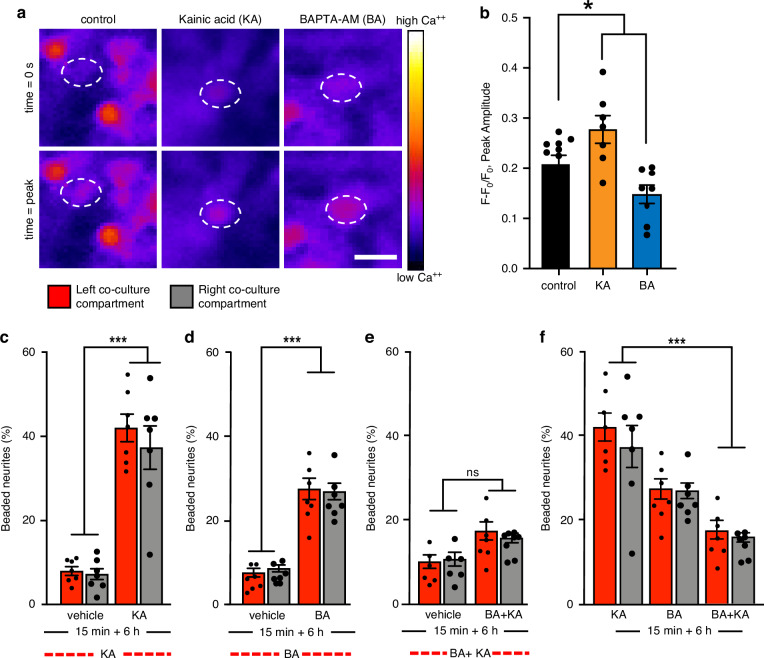
BAPTA-AM (BA) attenuated the intracellular calcium changes in astrocytes in response to KA in the left co-culture compartment and prevented neurite beading. **a** Fluo-4-direct (*F* − *F*_0_/*F*_0_) in astrocytes of the astrocytes-only compartment at baseline (time = 0), and maximum (time = peak), representing calcium changes following vehicle, KA or BA treatment. Scale bar = 25 µm. **b** Changes in fluo-4-direct, representing free intracellular calcium (*F* − *F*_0_/*F*_0_) in intracellular calcium levels following KA treatment (15-min) to the left co-culture compartment, or BA (15-min) to the astrocyte-only compartment. *p < 0.05; one-way ANOVA with Tukey’s post hoc test. *n* = 3 (mean ± SEM). **c** Beaded axons (%) in a ROI (mm^2^) of each co-culture compartment at 6 h following KA treatment (15-min) to the left co-culture compartment ***p < 0.001; one-way ANOVA with Tukey’s post hoc test. *n* = 3 (mean ± SEM). **d** Beaded axons (%) in a ROI (mm^2^) of each co-culture compartment at 6 h following 15-min BA treatment to the astrocyte-only compartment. ***p < 0.001; one-way ANOVA with Tukey’s post hoc test. *n* = 3 (mean ± SEM). **e** Beaded axons (%) in a ROI (mm^2^) of each co-culture compartment at 6 h following 15-min BA treatment to the astrocyte-only compartment prior to KA treatment (15-min) to the left co-culture compartment. ns non-significant; one-way ANOVA with Tukey’s post hoc test. *n* = 3 (mean ± SEM). **f** Beaded axons (%) in a ROI (mm^2^) of each co-culture compartment in KA-treated co-cultures 6 h following BA treatment to the astrocyte-only compartment, and 6 h following BA treatment to the astrocyte-only compartment prior to KA treatment to the left co-culture compartment. ns non-significant; one-way ANOVA with Tukey’s post hoc test. *n* = 3 (mean ± SEM)

Excitatory neurotransmitters activate astrocytic pathways involving rapid, long-distance calcium signalling^22^. We employed the strengths of our microfluidic model to investigate whether astrocytic calcium responses to KA contribute to the propagation of excitotoxic pathology. Live imaging of astrocytic calcium dynamics was performed using the calcium indicator Fluo-4 Direct (Fig. 6a; and Fig. S2c–h) after treatment of the left co-culture compartment. Changes in intracellular calcium (*F* − *F*_0_/*F*_0_) were calculated over 2 min in the astrocyte-only compartment under 3 conditions: control (Fig. 6a; and Fig. S2c, d), KA treatment (Fig. 6a, b, and Fig. S2e, f), or treatment of the astrocyte-only compartment with the membrane-permeable calcium chelator, BA (1 µM) (Fig. 6a, b, and Fig. S2g, h). KA treatment in the left co-culture compartment was associated with a significant (*P* < 0.05) increase in astrocytic calcium flux (0.28 *F* − *F*_0_/*F*_0_ ± 0.03 peak amplitude; *P* < 0.05) in the astrocyte-only compartment, compared to control (0.21 *F* − *F*_0_/*F*_0_ 0.02 peak amplitude) (Fig. 6b). In contrast, BA significantly reduced calcium flux (0.15 *F* − *F*_0_/*F*_0_ ± 0.02 peak amplitude) in the astrocyte-only compartment (Fig. 6b). These results show that KA treatment to the left co-culture compartment increased astrocytic calcium flux in the astrocyte-only compartment, and that this response can be attenuated by the calcium chelator BA reduced astrocytic calcium levels.

### Use of fluidic isolation to determine cell-specific function in co-culture networks

We next examined how astrocytic calcium levels and neurite pathology were affected following KA treatment. BA (1 µM) was applied to the astrocyte-only compartment for 15 min before KA (1 mM) was added to the left co-culture compartment for 15 min. Media was then replaced, and neurite degeneration was quantified at 6 h post-treatment. Neurite degeneration was compared between KA treatment alone, BA treatment alone in the astrocyte-only compartment, and “BA + KA” treatment.

KA exposure caused significant (*P* < 0.001) neurite degeneration in the left co-culture compartment (42.00% ± 3.27 beaded neurites), compared with vehicle-treated controls (8.03% ± 1.04; Fig. 6c). BA treatment alone in the astrocyte-only compartment also induced significant (*P* < 0.001) neurite beading in the left co-cultures compartment (27.52% ± 2.56) compared to vehicle-treated cultures (7.05% ± 1.01) (Fig. 6d). Pre-treatment with BA significantly (*P* < 0.001) attenuated KA-induced neurite beading (17.35% ± 2.14) compared to KA treatment alone (42.00% ± 3.27) (Fig. 6e, f).

A similar protective effect of BA was observed in the right co-culture compartment. BA treatment led to significant (*P* < 0.001) neurite degeneration (26.92% ± 1.97) relative to vehicle controls (8.66% ± 0.81; Fig. 6d). However, the addition of BA prevented the transfer of pathology from the left to the right co-culture compartments. Neurite beading in the “BA + KA” group was comparable to vehicle-treated cultures in the left (17.35% ± 2.14 vs 10.07% ± 1.06) and right (15.68% ± 1.14 vs 10.66% ± 1.63) compartments (Fig. 6e). These results demonstrate that chelating astrocytic calcium mitigates KA-induced excitotoxicity and its propagation through astrocytes. Notably, chelation of astrocytic calcium without the addition of KA caused significant neurite pathology in co-cultured neurons, indicating that astrocytic calcium signalling supports normal neuronal function.

## Discussion

Although traditional culture has provided important insights regarding astrocyte biology and their interactions with neurons, there is a growing need to develop and adopt innovative technologies that more accurately model the complexity of neuronal networks and astrocyte function in health and disease. Microfluidic cell culture platforms have gained popularity for their ability in the construction of heterogeneous cell networks that facilitate the study of cell-to-cell and network communication. For example, microfluidic devices have been used to culture 2D^23^ and 3D^24^ models of neuromuscular junctions, and a model of the blood-brain barrier that facilitates cell-to-cell communication between endothelial cells, astrocytes, and pericytes^25^. A neuron and glial co-culture microfluidic device was developed by Shi and colleagues^6^ demonstrated the critical role of glial-neuron communication in the formation and stabilisation of synaptic contacts. Similarly, previous studies that have looked specifically at the communication between neurons and astrocytes by compartmentalising neuronal somas, while permitting neurite outgrowth and synapse formation onto astrocytes^26^.

In this study, we developed a three-compartment microfluidic platform that uncouples the roles of astrocytes and neurons in network communication and the spread of neuropathology, independent of neuron-to-neuron signalling. To support the proliferation of primary astrocytes with 10–20 µm cell bodies^27^ we designed maze-like structures spaced 50 µm apart. By 7 DIV, astrocytes formed a monolayer throughout the device, confirming that the maze-like structures did not impede astrocyte growth (Fig. 2).

Previous studies show that microtopographic features can guide neuronal processes. Dowell-Mesfin and colleagues^28^ demonstrated that pillar-like structures direct axonal growth in primary hippocampal neurons. Another study achieved 99% unidirectional axonal growth^29^ of patient-derived midbrain dopaminergic neurons using a “fine leaves” microchannel pattern. These findings support the use of topography to guide axon and dendrite growth. Building on these studies, we designed our maze-like structures to prevent synaptic connectivity between neurons in two co-culture compartments. Each bank, 1 through 8, reduced the passage of neurites by 37% (Fig. 2), and ten banks were sufficient to prevent any neurites from entering the astrocyte-only compartment at 10 DIV of neuron-only cultures. Notably, when co-cultured with astrocytes, neurons failed to penetrate beyond bank 5 after 7 DIV. Proteomic profiling at identified neuron- and astrocyte-specific proteins in the co-culture compartments, whereas the astrocyte-only compartment showed enrichment for astrocytic markers and absence of neuronal proteins, confirming effective compartmentalisation (Fig. 3). Unlike previous devices, our platform enables the functional isolation of neuronal populations, connected only through an astrocyte monolayer. This facilitates the study of cell-type-specific contributions to network behaviour and pathology propagation, in the absence of direct synaptic transmission between neurons.

We further assessed the device’s ability to facilitate pharmacological manipulation of individual cell populations to determine their specific roles in network function and communication. KA, a well-characterised experimental excitotoxin, was used to induce excitotoxic pathology in neurons and assess the platform’s application for controlled pharmacological manipulation. KA was selected due to the differential expression of its receptors on neurons and astrocytes^30^. Unlike conventional protocols that apply KA at 0.1 mM for 12–24 h in mature cultures^31,32^, we reduced treatment duration to the period of fluidic isolation (5 or 15 min), at a concentration of KA that was up to 10-fold higher than previous studies in primary cultures^31,32^. This adjustment is particularly relevant for cortical neurons at 7 DIV, which are relatively resistant to excitotoxicity, compared to more mature cultures, where higher doses are used^33–35^. Neurite beading was used as a quantitative measure of excitotoxic pathology, consistent with previous studies^36,37^. Evidence of neurite beading in both the left (treated) and right adjacent co-culture compartments suggests that excitotoxicity was transferred through the astrocyte-only compartment connecting the two neuronal populations. Given that the co-culture compartments were under fluidic isolation, these data provide compelling evidence for a role of astrocytes in the transfer of excitotoxic pathology. When astrocytes were omitted from the device, no significant increase in KA-induced neurite beading was evident in the right adjacent compartment (Fig. 4), indicating astrocytes are required for the intercompartmental spread of pathology.

It is conceivable that neurotoxic effects on the adjacent neuron/astrocyte co-culture may be due to direct actions of KA on astrocytes. However, our data suggests that neurons, rather than astrocytes, initiate astrocytic calcium signalling that spreads KA-induced pathology between segregated neuron populations (Fig. 5). This finding aligns with our observation of minimal acute astrocytic toxicity following KA exposure, fitting with previous reports of astrocyte vulnerability only after prolonged exposure to KA (beyond 16 h)^38,39^. The delayed vulnerability of astrocytes may reflect the time needed to transcribe and express the AMPA/ Kainate receptors in response to high levels of KA^40^. Despite this, Cornell-Bell and colleagues^41^ reported that the acute application of KA (100 µM) to cultured astrocytes caused a transient rise in intracellular calcium that did not result in subsequent oscillations or the propagation of calcium waves through astrocyte monolayers. The data indicate that astrocytic calcium elevation, in isolation, is insufficient to propagate excitotoxic pathology.

Despite limited evidence that KA-induced excitotoxicity is directly transmitted through astrocytes, a small number of studies have detailed a role for glutamate-induced astrocytic activation in mediating neuronal excitotoxicity under pathological conditions such as status epilepticus^42,43^ and brain injury^44^. Astrocytes are implicated in the spread of neuronal excitotoxic pathology through calcium signalling and glutamate release^43^. For example, Robertson and colleagues used a microfluidic device to demonstrate that 0.1 mM glutamate can stimulate the transfer of calcium events to both neurons and astrocytes through synaptic connections^45^. Whilst the mechanistic association between calcium elevations in astrocytes and neuronal excitotoxicity is not fully resolved, Ding and colleagues have used calcium uncaging technology to show that elevations in astrocytic calcium can activate kainate receptors on adjacent neurons^46^. In an in vitro stroke model employing neuron–astrocyte co-cultures, Iwabuchi and colleagues^47^ further described a role for astrocytes in delayed neuronal death following acute excitotoxicity caused by glutamate uncaging. Collectively, these findings support a model in which neuronal excitotoxicity initiates calcium signalling in astrocytes, which may subsequently contribute to delayed or progressive neuronal damage.

The compatibility of our device with live-cell imaging techniques enabled further exploration of the role of astrocytic calcium in the spread of excitotoxic pathology between neuron populations. Calcium chelation in astrocytes is known to significantly reduce neuronal network synchronisation in organotypic hippocampal slices^48^. Specifically, calcium buffering in astrocytes reduces correlated activity of neurons, leading to the dysfunction of organised neuronal networks^49^. In our culture model, this is reflected by a marked increase in neurite beading following the application of the calcium chelator BA to the astrocyte-only compartment. Although BA treatment alone elevated baseline neurite beading, our data confirm that astrocytic calcium chelation can prevent KA-induced excitotoxic pathology and its transfer to synaptically segregated neuron populations (Fig. 6). These findings are consistent with previous reports demonstrating the neuroprotective effects of calcium chelation following calcium overload^50^ or glutamate-induced neurotoxicity^51^. Future studies should investigate whether astrocytes mediate excitotoxicity transfer through calcium signalling by incorporating gap-junction blockade^52,53^ and quantifying Ca²⁺ propagation in terms of waveform, velocity, and spatial correlation.

The presence of astrocytes in our culture model potentiated neurite pathology following KA treatment in co-cultures (Fig. 4 and Fig. S5). The mechanism underlying this association is likely related to the established role of astrocytes in promoting synaptogenesis and the maturation of excitatory synapses^54–56^. Several studies have demonstrated that astrocytes enhance glutamatergic synapse density and function in vitro^57–59^ and in vivo^57^. The astrocyte-mediated increase in functional excitatory synapses may contribute to the heightened susceptibility of neurons to KA susceptibility between neuron-enriched cultures, and neurons grown in co-culture^60^. Additional evidence from Vandenberghe and colleagues^61^ indicates that astrocytes can increase the vulnerability of primary motor neurons to KA through upregulation of Ca^2+^-permeable AMPA/kainite receptors. Although a detailed exploration into the mechanisms through which astrocytes confer neuronal vulnerability is beyond the scope of this study, we have established a platform that facilitates future molecular investigation of the role of astrocytes in neuron-to-neuron communication.

In this proof-of-principle study, we evaluated the effect of a single concentration of KA on astrocyte–neuron co-cultures under conditions that excluded direct neuron–neuron signalling using our microfluidic device. Future investigations applying a range of KA concentrations and exposure durations to neuron- or astrocyte-specific cultures within this platform will be critical for advancing drug discovery efforts targeting excitotoxicity-related neurological disorders. Nevertheless, our microfluidic platform supports a wide range of experimental applications in neuroscience research. It accommodates multiple CNS cell populations, including specific subtypes of neurons, astrocytes, microglia and oligodendrocytes. These cell types can be cultured either in isolation or in defined co-culture networks, facilitating the study of cell–cell interaction across physiological and pathological conditions. The device enables spatially controlled pharmacological manipulation, demonstrated in the current study by selective application of KA and BA to select cell populations. Its compatibility with immunocytochemistry, proteomic analysis, and live-cell imaging facilitates precise analysis of cellular responses to their microenvironment. This integrated approach offers a powerful tool for modelling disease-relevant conditions and probing the mechanisms by which localised insults impact neighbouring cells through intercellular signalling.

To expand the functionality of our platform, future iterations may incorporate a microvalved-controlled simulation channel^62^ adjacent to the astrocyte-only compartment. This addition would permit the collection and characterisation of astrocyte-released neuropeptides via mass spectrometry, providing a means to investigate whether astrocytes confer neuroprotection by releasing or transporting neuropeptides in response to excitotoxic stimuli. Our platform can also be integrated with advanced analytical techniques, such as single-cell microarrays and graphene oxide quantum dot (GOQD)-based assays for small extracellular vesicle profiling^63^ as well as single-cell sequencing^64,65^. In addition, the system offers a unique opportunity to evaluate the therapeutic potential of genetically engineered chimeric antigen receptor (CAR) T cells against target cancer cells^66^ within a controlled microenvironment. Furthermore, replacing primary cells with more physiologically relevant models, such as human induced pluripotent stem cell (hiPSC)-derived cell populations or cells from transgenic mice, will enhance disease modelling capabilities. These advances will enable a more detailed exploration of cell-type–specific contributions to neurodegenerative processes, with the goal of uncovering early mechanisms of disease progression mediated by cell–cell communication.

## Conclusion

We have developed a microfluidic co-culture device that enables physical and fluidic compartmentalisation of two primary neuronal populations, connected via astrocytes. The maze-like structure permits astrocyte infiltration while excluding neurites, allowing targeted manipulation and analysis of individual cell populations. Using this platform, we demonstrate that astrocytes can mediate excitotoxic pathology between segregated neurons, which likely involves a calcium-dependent mechanism. Our findings further highlight the ability of microfluidic systems to control synaptic connectivity and precisely manipulate the microenvironment of distinct cell populations. The platform’s capability for localised delivery of therapeutic agents, as demonstrated by excitotoxin screening, underscores its potential for studying cellular responses to spatially restricted chemical stimuli. We anticipate that this model will serve as a valuable tool for dissecting astrocyte-specific contributions to neuronal network dysfunction and for supporting the development of glia-targeted therapeutic strategies for CNS disorders.

## Supplementary information

Supplementary Figures and Figures legend

## Data Availability

All data are available in the main text or the supplementary materials.
