# Supplementary material for: Microfluidic co-culture system for synaptically segregated neural networks to explore astrocyte-driven neural pathology
Source: Microsyst Nanoeng. 2026 May 14;12:181. doi: 10.1038/s41378-026-01187-3 (PMC13172567; doi:10.1038/s41378-026-01187-3)
Supplement: Supplementary file 1 — Supplementary Figures and Figures legend [file 41378_2026_1187_MOESM1_ESM.docx]

**Supplementary Materials:**

­

­­­­­
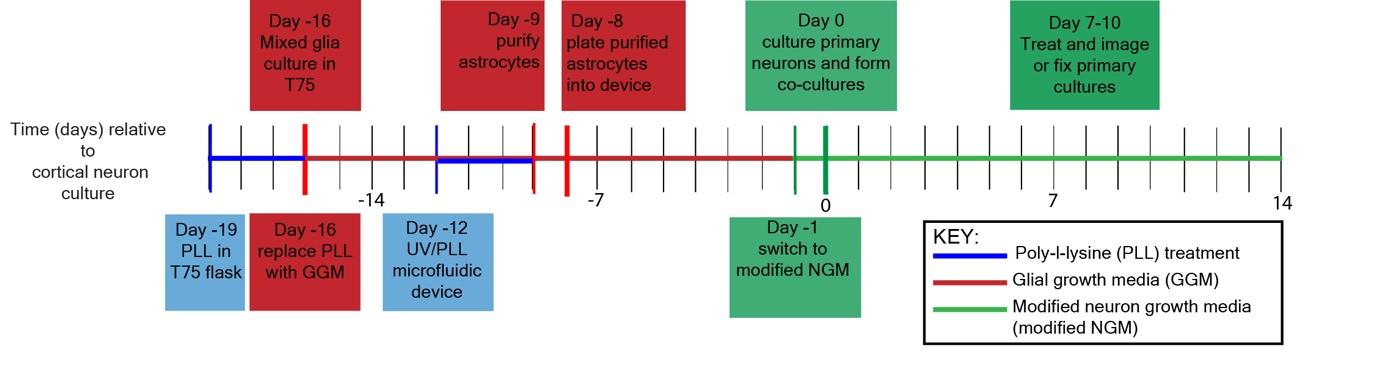


**Supplementary Fig. 1.** Timeline in days, for the preparation of the microfluidic device, the derivation, culture and plating of primary astrocytes and neurons, and maintenance of co-cultures in this microfluidic device.

**
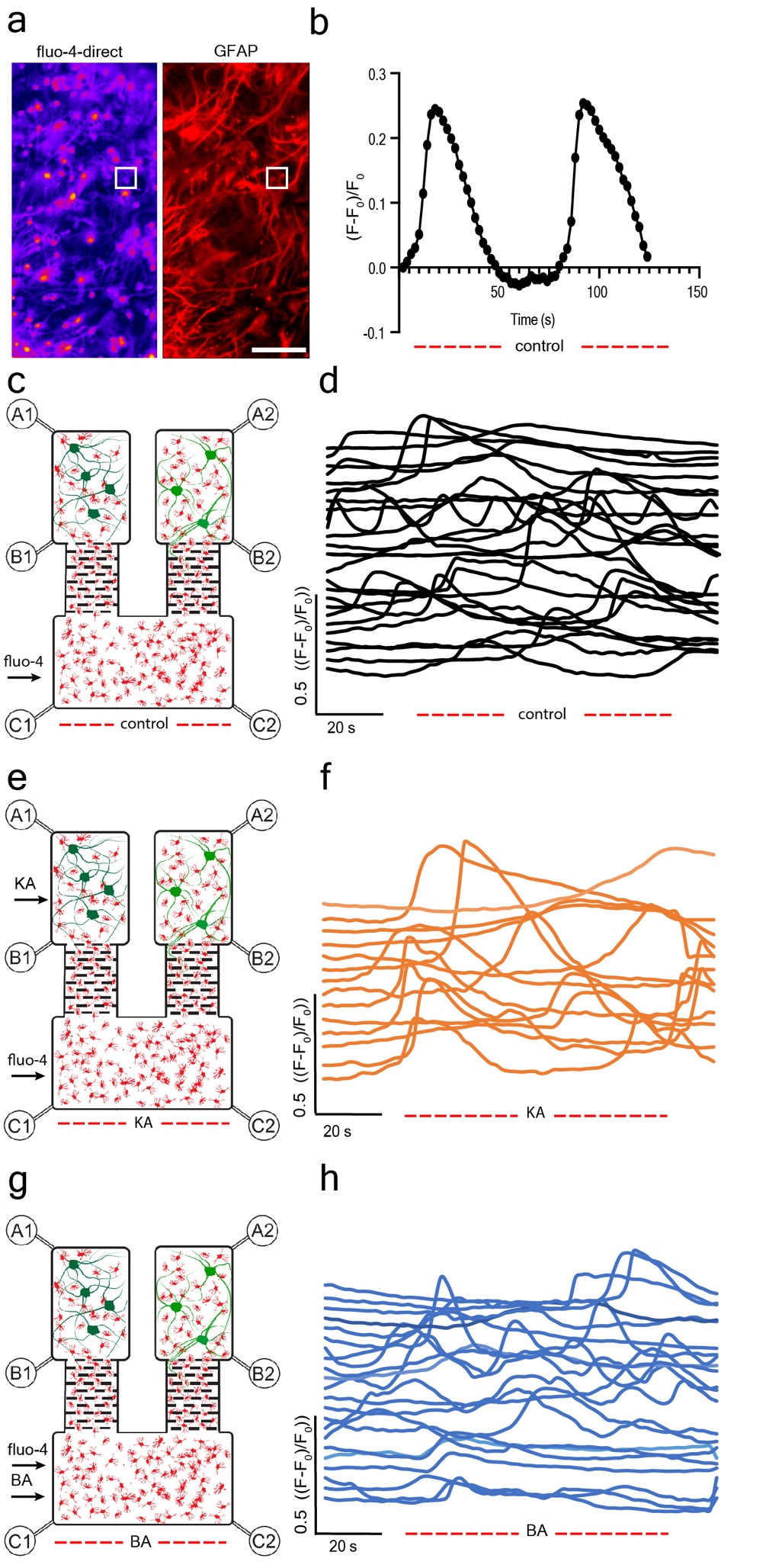
**

**Supplementary Fig. 2.** Effect of KA (1 mM) or BA (1 µM) on intracellular free calcium in astrocytes, measured with fluo-4-direct.

1. Fluo-4-direct in live untreated astrocytes, and GFAP immunolabelling of the same astrocyte-only compartment. White box indicates ROI around an individual astrocyte soma. Scale bar = 20 µm.
2. Dynamic changes in free intracellular calcium over time in the single untreated astrocyte soma from the ROI outlined in (g).
3. Schematic diagram of microfluidic device populated with astrocytes and neurons. Arrows indicate addition of fluo-4-direct to the astrocyte-only compartment.
4. Changes in fluorescence intensity of fluo-4-direct (F-F_0_/F_0_) over time, representing dynamic changes in free intracellular calcium levels in individual astrocytes of the astrocyte-only compartment.
5. Schematic diagram of microfluidic device populated with astrocytes and neurons. Arrows indicate addition of KA (1 mM) to the left co-culture compartment, and addition of fluo-4-direct to the astrocyte-only compartment.
6. Changes in fluorescence intensity of fluo-4-direct (F-F_0_/F_0_) in response to the addition of KA (1 mM) to the left co-culture compartment.
7. Schematic diagram of microfluidic device populated with astrocytes and neurons. Arrows indicates addition of fluo-4-direct and subsequently BA, to the astrocyte-only compartment.
8. Changes in fluorescence intensity of fluo-4-direct (F-F_0_/F_0_) in response to the addition of BA (1 µM) to the astrocytes-only compartment.


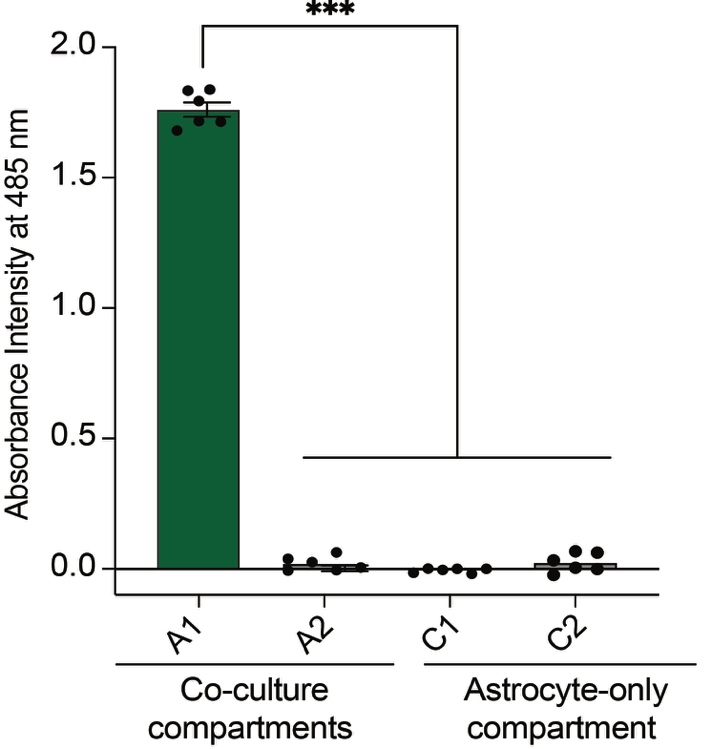


**Supplementary Fig. 3.** Absorbance measurements of fluorescent sodium salt (FI) were taken from reservoirs A1, A2, C1, and C2. FI was introduced into reservoir A1 of the left co-culture compartment for 15 minutes under fluidic isolation. The absorbance in reservoir A1 (1.763 ± 0.028) was significantly higher than in reservoirs A2, C1, and C2 (**p < 0.001, unpaired two-tailed Student’s t-test). Data are presented as mean ± SEM; n = 3 independent experiments.

**
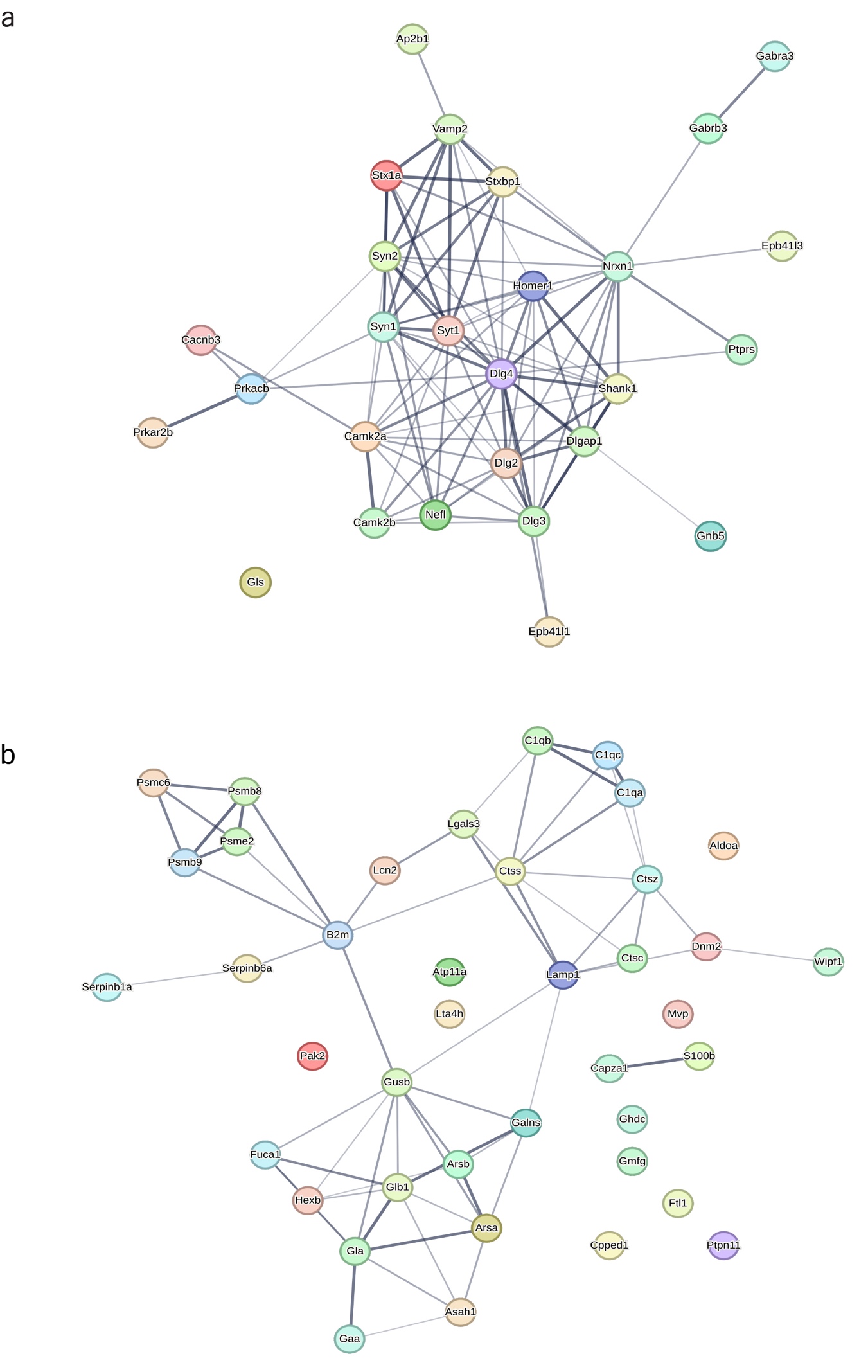
**

**Supplementary Fig. 4.** String analysis of the Reactome pathway of the cellular populations in different compartments of the microfluidic device.

1. STRING interaction network of significantly enhanced proteins expressed (-log_10_P value ≥5) in the co-culture compartments at 7 DIV that involved with neurotransmitter receptors, transmission across chemical synapse, and neuronal system.
2. STRING interaction network of significantly enhanced proteins expressed (-log_10_P value ≥5) in the astrocyte-only compartments at 7 DIV that involved in innate immune system and neutrophil degranulation.


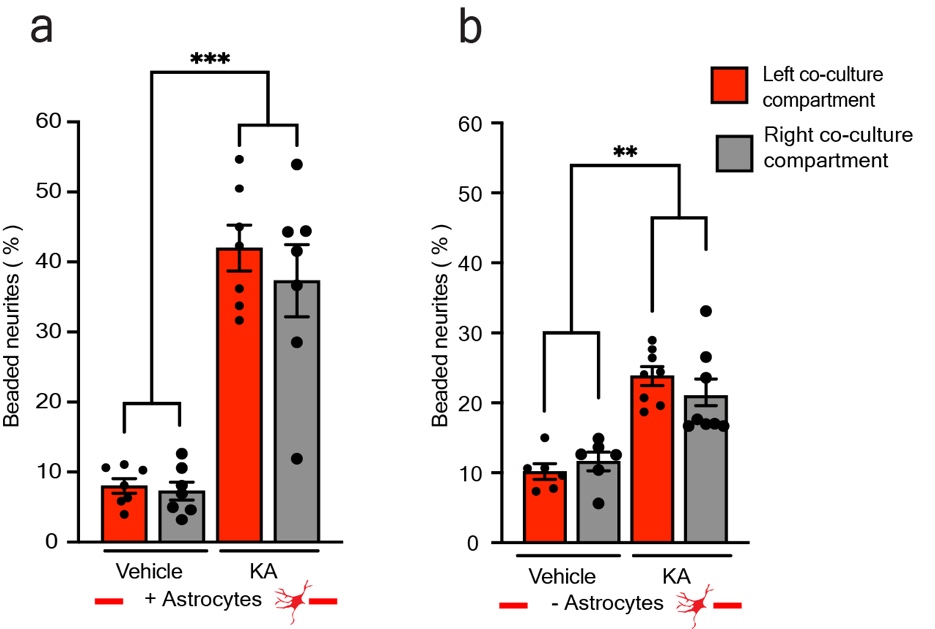


**Supplementary Fig. 5.** Kainic Acid (KA) treatment to the left co-culture compartment for 15 minutes caused a substantial loss of neurite integrity in neurons of the left and right co-culture compartments at 6 hours post-treatment in the presence of astrocytes.

1. Percentage of beaded axons in a mm^2^ ROI in the left and right co-culture compartments at 6 hours, following a 15-minute KA treatment to the left co-culture compartment in the presence of astrocytes; ***p<0.001; one-way ANOVA with Tukey’s post hoc test. Values represent mean ± SEM; n=3.
2. Percentage of beaded axons in a mm^2^ ROI in the left and right co-culture compartments at 6 hours, following a 15-minute KA treatment to the left co-culture compartment in the absence of astrocytes; **p<0.01; one-way ANOVA with Tukey’s post hoc test. Values represent mean ± SEM; n=3.

**Table S1:** Proteins significantly (-log_10_P value ≥ 5) enriched in the astrocyte-only compartments identified via DAVID Reactome pathway analysis. Proteins are clustered by function in the innate immune system and neutrophil granulation.

| Innate immune system | | Neutrophil degranulation | |
| --- | --- | --- | --- |
| Asah1 | Hexb | Fuca1 | Ctss |
| Ftl1 | Serpbinb1a | Ctsz | Lamp1 |
| Lta4h | Lgals3 | Lcn2 | Lta4h |
| Gaa | Gusb | Atp11a | Gmfg |
| Gmfg | Psmc6 | Asah1 | Lgals3 |
| Atp11a | Cpped1 | Serpinb1a | hexb |
| Psmb9 | C1qb | Aldoa | Ctss |
| C1qa | Serpinb6a | Gla | Lamp1 |
| C1qc | Psmb8 | Gusb | Lta4h |
| C1qb | Arsa | Gaa | Gmfg |
| Lamp1 | Ctsz | Serpinb6a | Arsb |
| B2m | Ptpn11 | Cpped1 | Galns |
| Wipf1 | Psme2 | Mvp | B2m |
| S100b | Mvp | Glb1 | Ctsc |
| Lcn2 | Aldoa | Lgals3 | Arsa |
| Glb1 | Ghdc | hexb | Ghdc |
| Arsb | Fuca1 |  |  |
| Pak2 | Ctss |  |  |
| Capza1 | Ctsc |  |  |
| Gla | Dnm2 |  |  |

**Table S2:** Proteins significantly (-log_10_P value ≥ 5) enriched in the co-culture compartments identified via DAVID Reactome pathway analysis. Proteins are clustered by involvement in neuronal system, the chemical synapse, and the Neurotransmitter Receptor.

| Nervous System | Synaptic transmission | Post-synaptic receptors |
| --- | --- | --- |
| Stx1a | Gnb5 | Dlg2 |
| Dlg3 | Stx1a | Camk2a |
| Dlgap1 | Camk2a | Gabra3 |
| Gabrb3 | Nefl | Prkar2b |
| Camk2a | Vamp2 | Dlg4 |
| Gabra3 | Epb4111 | Epb41l4 |
| Vamp2 | Prkacb | Dlg3 |
| Syn1 | Syt1 | Gabrb3 |
| Ep41l3 | Camk2b | Ap2b1 |
| Ap2b1 | Prkar2b | Mef2c |
| Gnb5 | Syn2 | Prkacb |
| Dlg4 | Cacnb3 | Gnb5 |
| Prkacb | Syn1 | Nefl |
| Dlg2 | Dlg4 | Camk2b |
| Nrxn1 | Ap2b1 |  |
| Epb41l1 | Gabra3 |  |
| Ptprs | Dlg3 |  |
| Homer1 | Stx1bp1 |  |
| Camk2b | Gls |  |
| Prkar2b | Dlg2 |  |
| Stxbp1 | Gabrb3 |  |
| Gls |  |  |
| Syn2 |  |  |
| Syt1 |  |  |
| Shank1 |  |  |
| Cacb3 |  |  |
| Nefl |  |  |
|  |  |  |
|  |  |  |
|  |  |  |
|  |  |  |
|  |  |  |
